# Crystal structures of ‘ALternative Isoinformational ENgineered’ DNA in B-form

**DOI:** 10.1098/rstb.2022.0028

**Published:** 2023-02-27

**Authors:** Madhura S. Shukla, Shuichi Hoshika, Steven A. Benner, Millie M. Georgiadis

**Affiliations:** ^1^ Department of Biochemistry and Molecular Biology, Indiana University School of Medicine, Indianapolis, IN 46202, USA; ^2^ Foundation for Applied Molecular Evolution, 13709 Progress Boulevard, no. 7, Alachua, FL 32615, USA

**Keywords:** ALIEN DNA, crystal structures, B-form DNA, xNA, unnatural DNA

## Abstract

The first structural model of duplex DNA reported in 1953 by Watson & Crick presented the double helix in B-form, the form that genomic DNA exists in much of the time. Thus, artificial DNA seeking to mimic the properties of natural DNA should also be able to adopt B-form. Using a host–guest system in which Moloney murine leukemia virus reverse transcriptase serves as the host and DNA as the guests, we determined high-resolution crystal structures of three complexes including 5′-CTT**BPPBBSSZZS**AAG, 5′-CTT**SSPBZPSZBB**AAG and 5′-CTT**ZZPBSBSZPP**AAG with 10 consecutive unnatural nucleobase pairs in B-form within self-complementary 16 bp duplex oligonucleotides. We refer to this ALternative Isoinformational ENgineered (ALIEN) genetic system containing two nucleobase pairs (**P:Z**, pairing 2-amino-imidazo-[1,2-*a*]-1,3,5-triazin-(8*H*)-4-one with 6-amino-5-nitro-(1*H*)-pyridin-2-one, and **B:S**, 6-amino-4-hydroxy-5-(1*H*)-purin-2-one with 3-methyl-6-amino-pyrimidin-2-one) as ALIEN DNA. We characterized both position- and sequence-specific helical, nucleobase pair and dinucleotide step parameters of **P:Z** and **B:S** pairs in the context of B-form DNA. We conclude that ALIEN DNA exhibits structural features that vary with sequence. Further, **Z** can participate in alternative stacking modes within a similar sequence context as captured in two different structures. This finding suggests that ALIEN DNA may have a larger repertoire of B-form structures than natural DNA.

This article is part of the theme issue ‘Reactivity and mechanism in chemical and synthetic biology’.

## 1. Introduction

DNA serves as the genetic storage system for all Terran life. And, it is the sequence of the DNA that determines its properties. These sequence-specific properties allow proteins to discriminate among DNA sequences and contribute to the ability of proteins to recognize, bend or otherwise alter the form of duplex DNA [1,2]. The ability to define properties of specific DNA sequences relies on structural analysis as demonstrated in the first crystal structure of B-form DNA [3]. An open question asks: Are the properties of natural DNA distinct from those of ALternative Isoinformational ENgineered (ALIEN) DNA in its B-form? In this study, we characterized the properties of ALIEN DNA containing 10 consecutive unnatural **P:Z** and **B:S** nucleobase pairs in B-form in three high-resolution crystal structures.

**Figure 1.**
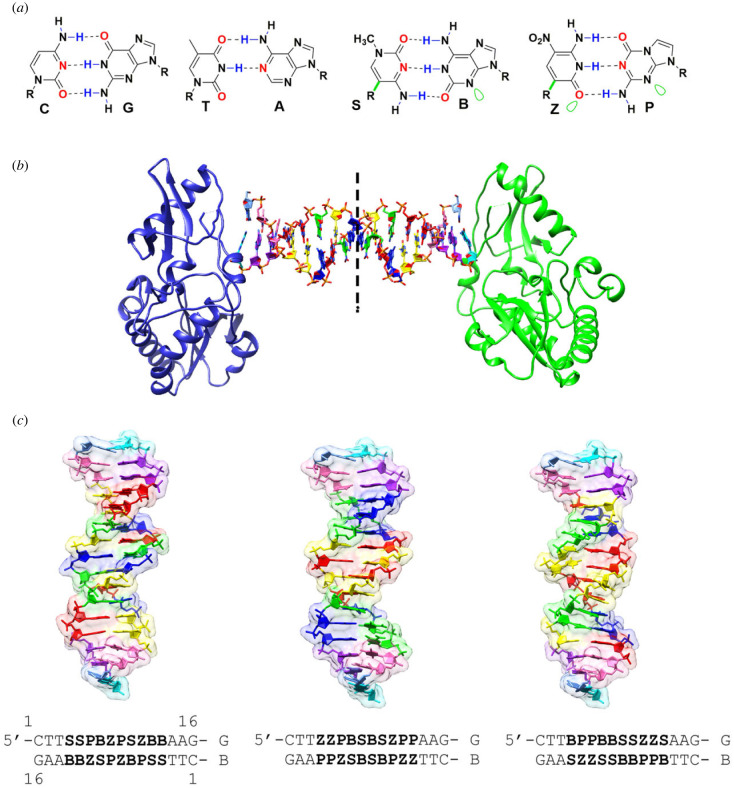
Crystal structures of ALIEN DNA containing 10 consecutive unnatural nucleobase pairs. (*a*) Structures of unnatural nucleobases found in ALIEN DNA are shown; green bonds are *C*-glycosides. Green lobes indicate electron density presented to the minor groove. (*b*) The crystal structures of ALIEN DNA were obtained in host–guest complexes. The host is the N-terminal fragment of Moloney murine leukemia virus reverse transcriptase and the guest is a self-complementary 16 bp oligonucleotide duplex. The host–guest complex includes one protein molecule (shown as blue and green cartoon renderings) bound on each end of the 16 bp duplex DNA. The DNA shown is the **SSPBZ** sequence, with **Z** green, **P** blue, **B** red, **S** yellow, A purple, T pink, G cyan and C light blue. Half of this complex is in the asymmetric unit of the crystal. (*c*) The three ALIEN DNA structures are shown with a transparent molecular surface; the sequences are indicated below. The colour scheme is the same as indicated in (*b*). The numbering for each oligonucleotide strand is indicated in (*c*) and the chain identifiers for each strand. (Online version in colour.)

ALIEN DNA in this study includes the **P:Z** pair between 2-amino-imidazo-[1,2-*a*]-1,3,5-triazin-(8*H*)-4-one with 6-amino-5-nitro-(1*H*)-pyridin-2-one, and the **B:S** pair between 6-amino-4-hydroxy-5-(1*H*)-purin-2-one with 3-methyl-6-amino-pyrimidin-2-one [4]. Three hydrogen bonds that join the **P:Z** and **B:S** pairs are stereochemically distinct from each other (figure 1*a*) and from those of standard nucleobase pairs. In order to achieve these distinct hydrogen-bonding patterns for **P:Z** and **B:S**, both **Z** and **S** must be attached to the deoxyribose sugar by a carbon–carbon bond (a *C*-glycoside, figure 1*a*, green bond), rather than the carbon–nitrogen bond (*N*-glycoside) that attaches natural nucleotides to their carbohydrate backbones.

Other properties distinguish these unnatural base pairs from natural base pairs. First, to prevent oxidation and/or epimerization of **Z**, an electron-withdrawing group must be present at its ‘5-position’. A nitro group serves this purpose. However, the nitro group effectively makes this nucleobase larger than standard pyrimidine nucleobases. Second, the nucleobase **S** must present a hydrogen-bond donor (rather than acceptor) to the minor groove, in contrast with all standard nucleobases and the other non-standard nucleobases used in this study, in order to achieve the desired hydrogen-bonding with **B** [5].

In this study, we determined crystal structures for three ALIEN DNA sequences through the use of our host–guest system, in which the N-terminal fragment of Moloney murine leukemia virus reverse transcriptase serves as the host and a self-complementary 16 bp duplex as the guest [6,7]. There are several advantages to using this host–guest system. These include the ability to phase the crystal structures using the protein molecule as the search model in molecular replacement calculations; this results in unbiased electron density for the DNA. They also include the ability to analyse the properties of the DNA sequences in the same crystal lattice, thereby eliminating lattice-specific effects.

Using this host–guest system, we have determined over 30 crystal structures of different natural DNA sequences in B-form ([6,8–23]; electronic supplementary material). Here, we analysed both position-specific and sequence-specific local base pair and base pair step properties and compared these properties in our ALIEN DNA and natural DNA structures determined in the same host–guest crystal lattice.

## 2. Results and discussion

### (a) Crystal structures of ALternative Isoinformational ENgineered DNA as host–guest complexes

To capture ALIEN DNA in B-form, we used our host–guest system to crystallize self-complementary oligonucleotides, 5′-CTT**BPPBBSSZZS**AAG (**BPPBB**), 5′-CTT**SSPBZPSZBB**AAG (**SSPBZ**) and 5′-CTT**ZZPBSBSZPP**AAG (**ZZPBS**). We have also determined the structure of 5′-CT**SZZPBSBSZPPB**AG (**SZZPBS**) as a host–guest complex. This structure is reported in detail elsewhere [24] in a comparison with the same sequence crystallized without the host; it is included here as part of our comparative analysis. In our experience, if a sequence can adopt a form similar to B-form DNA, it will crystallize as a host–guest complex ([16] and electronic supplementary material). Interactions between the protein and DNA are limited to hydrogen-bonding interactions of Arg 116 with the minor groove of the terminal base pairs within the duplex, leaving the remainder of the duplex free to adopt its preferred sequence-specific structure [7].

As the sequence on the ends of the duplex is the same in each of the structures and includes 5′-CTT, the interactions of the protein with the DNA are nearly identical (electronic supplementary material, figure S1). As in most of the host–guest complexes determined to date, the asymmetric unit includes one protein molecule and half of the 16 bp duplex. The structures were determined by molecular replacement phasing obtained for the protein alone, and the DNA model was fitted into unbiased electron density in COOT [11,25]. We refined the structures using PHENIX [12,26] (table 1). Although the host–guest system offers advantages for crystallization, phasing and analysis in the same lattice, it imposes some restrictions on the structure of the DNA dictated by interactions of the terminal 2 or 3 nucleobase pairs with the host and the forces governing the crystal lattice. This latter limitation is present in all crystal structures.

**Table 1.** Crystallographic data.

	SSPBZ	ZZPBS	BPPBB
PDB ID	7UYO	7UYP	7UYN
**data collection**			
space group	P21212	P21212	P21212
cell dimensions			
*a*, *b*, *c* (Å)	55.31, 145.93, 46.90	54.98, 145.76, 46.96	54.85, 146.48, 46.66
*α*, *β*, *γ* (°)	90, 90, 90	90, 90, 90	90, 90, 90
wavelength (Å)	0.97933	0.97933	0.97933
resolution (Å)	145.93–1.65 (1.68–1.65)	72.88–1.51 (1.59–1.51)	73.24–1.65 (1.68–1.65)
*R*_merge_	0.048 (0.792)	0.048 (0.551)	0.098 (1.315)
*R*_pim_	0.0022 (0.352)	0.022 (0.252)	0.029 (0.408)
CC(1/2)	0.999 (0.767)	0.999 (0.876)	0.998 (0.697)
mean *I*/*σ*(*I*)	17.0 (2.2)	17.4 (2.7)	14.7 (2.3)
completeness (%)	99.8 (99.9)	99.7 (98.8)	99.9 (99.9)
redundancy	6.5 (6.7)	6.4 (6.4)	12.5 (11.6)
**refinement**			
resolution (Å)	27.652–1.65	27.489–1.51	30.46–1.65
no. reflections	46 443	60 881	46 128
*R*_work_/*R*_free_ (%)	0.227/0.256	0.216/0.235	0.222/0.246
no. atoms/*B*_ave_			
protein	1982/37.41	1994/32.16	1989/29.05
DNA	334/71.29	336/62.59	334/59.42
water	154/36.57	190/35.34	154/30.29
r.m.s. deviation			
bond lengths (Å)	0.006	0.006	0.007
bond angles (°)	1.001	0.930	1.049

These are the first structures to include more than six consecutive unnatural **P:Z** and **B:S** pairs (figure 1*a*) as in the 1.7 Å hachimoji structure of 5'-CTTAT**PPSBZZ**ATAAG [13]. All of these host–guest structures include 5′-CTT in positions 1–3 (14–16 on the complementary strand) and 5′-**PB** at positions 6 and 7 (11 and 10) in the oligonucleotides (figure 1*b,c*). The sequence in the other positions within the duplex differs and provides an opportunity to examine the effects of flanking unnatural nucleobase pairs on the structure of ALIEN DNA. Both **SSPBZ** and **ZZPBS** structures offer a pattern of two pyrimidine, two purine and a pyrimidine nucleobase. The **BPPBB** structure includes a tract with five consecutive purines (or five pyrimidines on the complementary strand), offering the first view of a purine-rich tract in ALIEN DNA (figure 1*c*).

As in most of our host–guest complexes, there are a large number of solvent molecules bound to the host protein and very few DNA-bound solvent or counter-ions. Within these three structures, five water molecules were identified that directly contact the DNA (electronic supplementary material, figure S2). All were modelled as water molecules in the absence of any experimental evidence for the presence of counter-ions. Of these, three were identified in the **ZZPBS** structure. Two make nucleobase-specific contacts, and one a backbone contact. Of the nucleobase contacts in the **ZZPBS** structure, one water is located 2.7 Å from N3 of A15 and 2.8 Å from O4′ of G16; the second is located 2.9 Å from N7 of G16. The water molecule associated with the backbone is 2.9 Å from OP1 of G16 and is hydrogen-bonded to two other water molecules, which are in turn hydrogen-bonded to other water molecules. There is a water molecule bound to the backbone along with associated water molecules present in each of the other structures as well.

### (b) ALIEN DNA in a host–guest complex is B-form

To determine the helical form of ALIEN DNA in our crystal structures, we used 3DNA [27–29] to obtain parameters from 29 host–guest DNA structures reported at resolutions of 2.35 Å or higher in the same crystal lattice as our new structure and 31 A-form DNA structures reported at 2 Å or higher resolution (electronic supplementary material, table S2). We plotted slide versus roll, *x*-displacement versus inclination and Zp versus ZpH, which are defined as the ‘projection of the phosphorus atom onto the *z*-axis of the dimer middle frame’ and the half distance of this projection, respectively [30]. In this context, ‘dimer’ refers to a dinucleotide step, and the coordinate frame is explained within [30].

**Figure 2.**
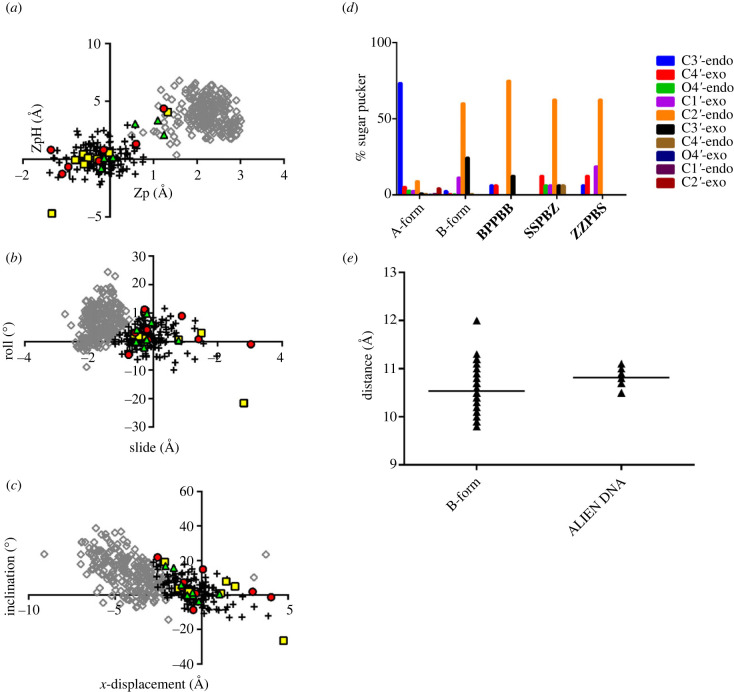
Helical form of ALIEN DNA. Zp versus ZpH (*a*), slide versus roll (*b*) and *x*-displacement versus inclination (*c*) were plotted for A-form (grey open diamonds) and B-form (black '+' symbols) nucleobase pairs from our reference database. Parameters from **ZZPBS** are plotted as green triangles, **SSPBZ** red circles and **BPPBB** yellow squares. ALIEN DNA can be classified as B-form based on this analysis. (*d*) The distributions of sugar pucker conformations are plotted for A-form, B-form and the three ALIEN DNA structures. The most common sugar pucker is C2′-endo as is commonly found in B-form DNA. (*e*) C1′–C1′ distances are plotted for reference B-form DNA structures and our ALIEN DNA structures. ALIEN DNA pairs include **Z** and **S**, which contain *C*-glycosidic linkages rather than *N*-glycosidic linkages. The average C1′–C1′ distances in ALIEN DNA are slightly longer than the average for natural B-form DNA. (Online version in colour.)

As previously reported [27], plotting parameters derived from B-form DNA results in a cluster of points that is distinguishable from a cluster for those same parameters derived from A-form DNA. In the Zp versus ZpH plot, most points fall with the B-form cluster (figure 2*a*), with five exceptions, one for **SSPBZ**, two for **BPPBB** and two for the **ZZPBS** structures. Four of these exceptions fall within the A cluster. In each structure, the terminal CT/AG dinucleotide step falls outside of the B cluster. The second **ZZPBS** point that falls outside of the structure is the **ZZ/PP** step (positions 4 and 5, referring to base pairs from the 5′ end). The second **BPPBB** point that falls outside the B cluster is the **BS/BS** step (positions 8 and 9). For slide versus roll (figure 2*b*), two points fall in the same quadrants of the graph as B-DNA but extend beyond these values; these points are the **SP/ZB** point for the **SSPBZ** structure, in which the slide value is 3.03 Å, and the **BS/BS** point for the **BPPBB** structure, with a slide value of 2.81 Å and a roll value of −21.52°. For the *x*-displacement versus inclination plot (figure 2*c*), most of the points fall within the B cluster. However, the value of the **BS/BS** step (position 8 and 9) of **BPPBB** falls out of the range, with an *x*-displacement of 4.73 Å and inclination value of −26.46°.

By these criteria, we classify the ALIEN DNA in these structures as B-form. Another parameter that distinguishes A- from B-form DNA is the pucker of the sugar ring. For all three structures, over 50% of the sugars adopt the C2′-endo conformation most commonly observed in B-form DNA (figure 2*d*). For comparison, 74% of sugars in A-form DNA are C3′-endo. Analysis of the C1′–C1′ distances for ALIEN base pairs indicates that, although the distances fall within the range seen in natural B-DNA (9.8–12 Å), the average distance of 10.8 Å is slightly longer in ALIEN base pairs than in natural base pairs, with an average of 10.5 Å; each ALIEN base pair includes a *C*-glycoside, **S** for the **S:B** pair and **Z** for the **Z:P** pair (figure 2*e*).

### (c) ALIEN DNA structures exhibit sequence-specific structural properties

**Figure 3.**
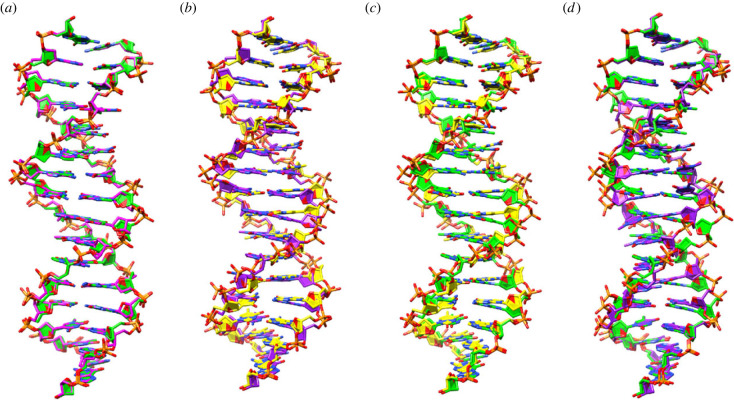
Comparison of ALIEN DNA structures. In (*a*) are two superimposed host–guest structures, **SZZPBS** (magenta) and **SSPBZ** (green) that are remarkably similar (r.m.s.d. 0.6 Å), especially given that they do not share the same sequence. They do share the same pattern of ALIEN nucleobases with two pyrimidines, two purines and a pyrimidine. The structures were superimposed in Chimera using Matchmaker [31]. (*b*) **SZZPBS** (magenta) and **ZZPBS** (yellow) are superimposed (r.m.s.d. 1.7 Å). Despite sharing the same sequence, these two structures are less similar to each other than those depicted in (*a*). (*c*) **SSPBZ** (green) and **ZZPBS** (yellow) (r.m.s.d. 1.8 Å) and (*d*) **SSPBZ** (green) and **BPPBB** (purple) (r.m.s.d. 1.67 Å) are shown superimposed. (Online version in colour.)

Superpositioning of the ALIEN DNA in the host–guest structures results in r.m.s.d. values ranging from 0.6 to 2.6 Å, with the most similar being **SSPBZ** and **SZZPBS** and the least similar being **BPPBB** and **ZZPBS** (figure 3; electronic supplementary material, table S1). It is not surprising that **BPPBB** differs from the other structures, since it contains five consecutive purine nucleobases; the other structures exhibit a pattern of two pyrimidine, two purine and a pyrimidine. What is surprising is that **BPPBB** is much more similar to **SSPBZ** with an r.m.s.d. of 1.67 Å than to **ZZPBS** (r.m.s.d. 2.6 Å). Despite having direct overlap of the same ALIEN sequence, **SZZPBS** and **ZZPBS** superimpose with an r.m.s.d. of 1.66 Å, whereas **SSPBZ,** which differs in sequence from **SZZPBS,** superimposes with an r.m.s.d. of 0.6 Å (figure 3). In fact, these two structures are the most similar structures that we have analysed to date. To determine underlying features that contribute to these structural differences, we examined nucleobase pair hydrogen-bonding, local base pair parameters and local base pair step parameters for all possible nucleobase pairs. We analysed these parameters for specific nucleobase pairs and dinucleotide steps; we also analysed the properties relative to the position in the oligonucleotide duplex.

Most of the nucleobase pairs, natural and unnatural, form the expected hydrogen bonds (figure 1). There are three exceptions. In the **SSPBZ** structure, the T:A pair at position three forms only a single hydrogen bond between N3 and N1, potentially owing to a large negative propeller angle (electronic supplementary material, figure S3*a*). Similarly, the **S:B** pair at position 3 in the **SZZPBS** structure forms only two (between N3 and N1 and N2 and O2) of the three possible hydrogen bonds and also exhibits a large negative propeller angle (electronic supplementary material, figure S3*b*). In the **BPPBB** structure, the **B:S** pair at position 4 forms only two hydrogen bonds between O2 of **B** and N2 of **S**, N1 of **B** and N3 of **S** (electronic supplementary material, figure S3*c*).

We next analysed local base parameters including shear, stretch, stagger, buckle, propeller and opening (electronic supplementary material, figure S4). We compared **P:Z** and **S:B** nucleobase pair parameters to those of A:T and G:C pairs. Values observed for all of the ALIEN local base parameters fall within the range observed for natural nucleobase pairs. However, we note that the range of these values is likely context-dependent, meaning that these parameters are influenced by neighbouring nucleobase pairs.

To determine position-specific effects for the local base pair parameters, we plotted ALIEN DNA parameters for each position of the nucleobase pair within the oligonucleotide relative to the end, as well as parameters for an AT-rich sequence (5′-CTTATAAATTTATAAG, PDB: 4XPC) and a GC-rich sequence (5′-CTTATGGGCCCATAAG, PDB: 4XPE) for previously reported structures. This is an alternative way to examine the sequence-specific effects and neighbour effects within each structure. Values for stretch and stagger were relatively small and uniform across the structures and were not further considered. Values for opening also showed similar trends across the structures.

**Figure 4.**
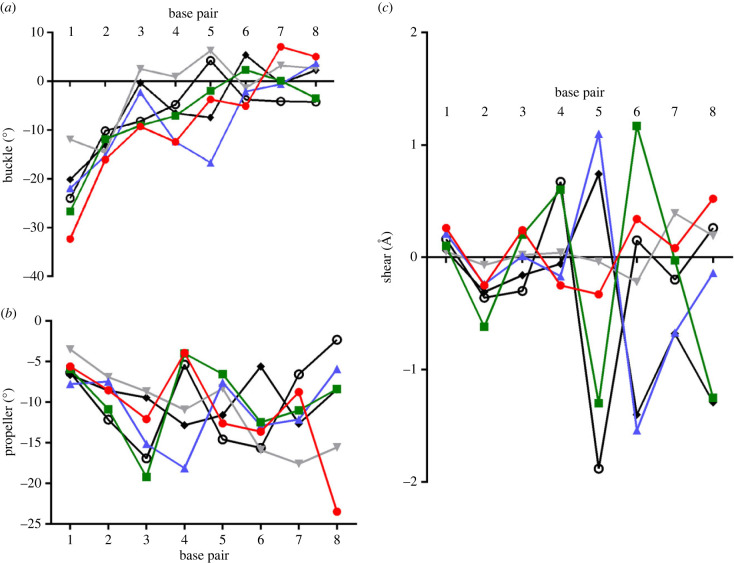
Position-specific analysis of local base pair parameters. Buckle angle (*a*), propeller angle (*b*) and shear distance (*c*) are plotted for each position in natural AT-rich, GC-rich and ALIEN DNA structures. Plots are shown for **BPPBB** (red circles), **SSPBZ** (green squares), **ZZPBS** (blue triangles), AT (grey upside-down triangles), GC (black filled diamonds) and **SZZPBS** (open black circles). Specific patterns for these parameters are observed in both **SZZPBS** and **SSPBZ**. (Online version in colour.)

Parameters in the ALIEN DNA structures that might account for observed structural differences include buckle, propeller and shear. The largest differences in buckle were observed in the **ZZPBS** structure for the **Z:P** pair at position 5 (figure 4*a*). This pair has a buckle angle of −16.7°. In all other structures, this angle falls between −4° and +6°. Propeller angles for **SSPBZ** and **SZZPBS** exhibit a similar pattern, with a large negative propeller angle at position 3, −19.2° and −16.9°, respectively, (T:A in **SSPBZ** and **S:B** in **SZZPBS**) and a much smaller negative angle at position 4, −4° and −5.4°(**S:B** and **Z:P** pairs). **SZZPBS** has a large negative angle at position 5, of −14.6° (figure 4*b*). By contrast, **ZZPBS** exhibits a larger negative angle at position 4 (−18.1°) for the **Z:P** pair and a smaller negative angle at position 5 (−7.6°) for the **Z:P** pair as compared with the same steps in the **SZZPBS** structure.

Perhaps the most distinguishing parameter is the shear value. Both **SSPBZ** and **SZZPBS** exhibit alternating shear values for positions 4–6, opposite in sign to those in the **ZZPBS** structure. At position 4, shear is 0.6 and 0.67 Å, at position 5, −1.3 and −1.9 Å, and at position 6, 1.17 and 0.15 Å in **SSPBZ** versus **SZZPBS** (figure 4*c*). In **ZZPBS**, shear values at positions 4–6 are −0.17, 1.10 and −1.54 Å, respectively. The pattern of shear values for **ZZPBS** most closely resembles that observed for the GC-rich sequence.

**Figure 5.**
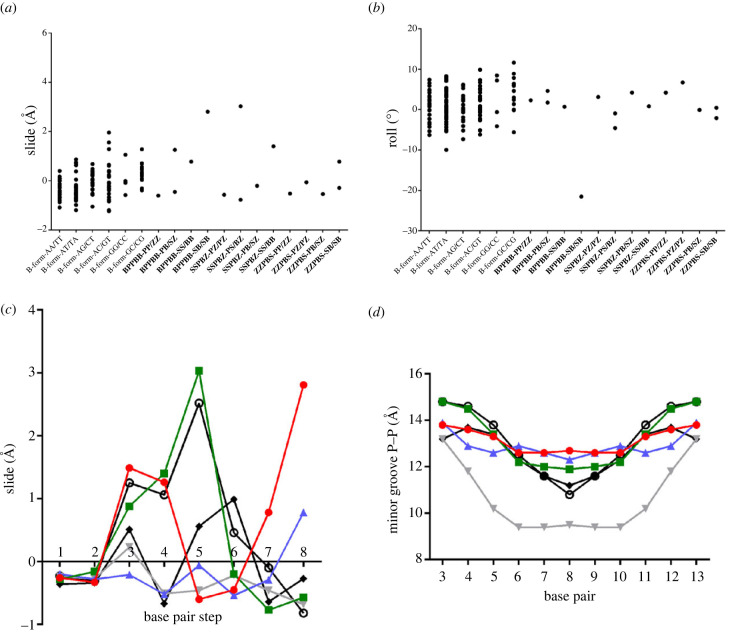
Analysis of dinucleotide step parameters. The range of values observed for natural and ALIEN dinucleotide steps are plotted for slide distance (*a*) and roll angle (*b*). ALIEN DNA structures exhibit some roll and slide values that fall outside of the variations observed for natural dinucleotide steps. Position-specific analysis of slide (*c*) reveals similarities in **SZZPBS** and **SSPBZ** structures. Minor groove widths in all of the ALIEN DNA structures are more similar to GC DNA than AT DNA, as expected (*d*). Minor grooves are somewhat wider in most of the ALIEN DNA structures. Plots in (*c*) and (*d*) are shown for **BPPBB** (red circles), **SSPBZ** (green squares), **ZZPBS** (blue triangles), AT (grey upside-down triangles), GC (black filled diamonds) and **SZZPBS** (open black circles). (Online version in colour.)

We next analysed base pair step parameters, including slide, rise, roll and twist for each type of dinucleotide step in ALIEN and natural DNA. Values for rise and twist all fall within the range observed for natural DNA (electronic supplementary material, figure S4*g*,*h*). However, two dinucleotide steps exhibit slide values outside of the natural range. **BS/BS** (positions 8 and 9) in the **BPPBB** structure has a slide value of 2.8 Å, and the **SP/ZB** step in the **SSPBZ** structure a value of 3.0 Å (figure 5*b*). The range observed in natural DNA extends from −1.2 to 2.0 Å. The same dinucleotide step, **BS/BS** in the **BPPBB** structure, has a roll angle of −21.5° (figure 5*a*). The range for roll angles in natural DNA is −10° to 11.7°. This is likely a consequence of the five consecutive purine nucleobases abutting five consecutive pyrimidines across the central dyad of the oligonucleotide. Slide values plotted by position in the oligonucleotide highlight the similarities in the **SSPBZ** and **SZZPBS** structures, with large positive slide values at position 5 of 3.0 and 2.5 Å, respectively. The **BPPBB** structure, as noted above, has a slide value of 2.8 Å at step position 8, a feature distinguishing it from the other structures (figure 5*c*).

Finally, we analysed the minor groove widths for each of the structures and compared them to the AT-rich and GC-rich sequences. At the narrowest point, the minor groove widths for all of the structures except **SZZPBS** are slightly wider than that observed for the GC-rich sequence. The AT-rich sequence has a minor groove width of 9.5 Å; in the GC-rich sequence, the width is 11.2 Å. Minor groove widths of 10.8, 11.9, 12.3 and 12.7 Å were observed for **SZZPBS**, **SSPBZ**, **ZZPBS** and **BPPBB**, respectively (figure 5*d*). Thus, all of the ALIEN DNA sequences are more similar to a GC-rich oligonucleotide than the AT-rich sequence in this regard.

## 3. Conclusion

ALIEN DNA exhibits sequence-specific variations in structure similar to natural DNA. Although **ZZPBS** includes the same sequence as **SZZPBS**, it is less similar in structure to **SZZPBS** than the **SSPBZ** structure. While **SZZPBS** and **SSPBZ** differ in sequence, the stacking interactions of the **Z:P** pairs in **SZZPB**S are remarkably similar to the **S:B** pairs in **SSPBZ** (figure 3*a*). In contrast with this finding, a contributing factor to the dissimilarity of **ZZPBS** and **SZZPBS** appears to be the stacking interaction of the **Z:P** pair at position 4 with the nucleobase pairs at position 3. In the **ZZPBS** structure, there is a T:A pair at position 3, while in **SZZPBS**, there is an **S:B** pair. In each of these structures, the **Z:P** pair at position 4 also stacks with a **Z:P** pair at position 5.

**Figure 6.**
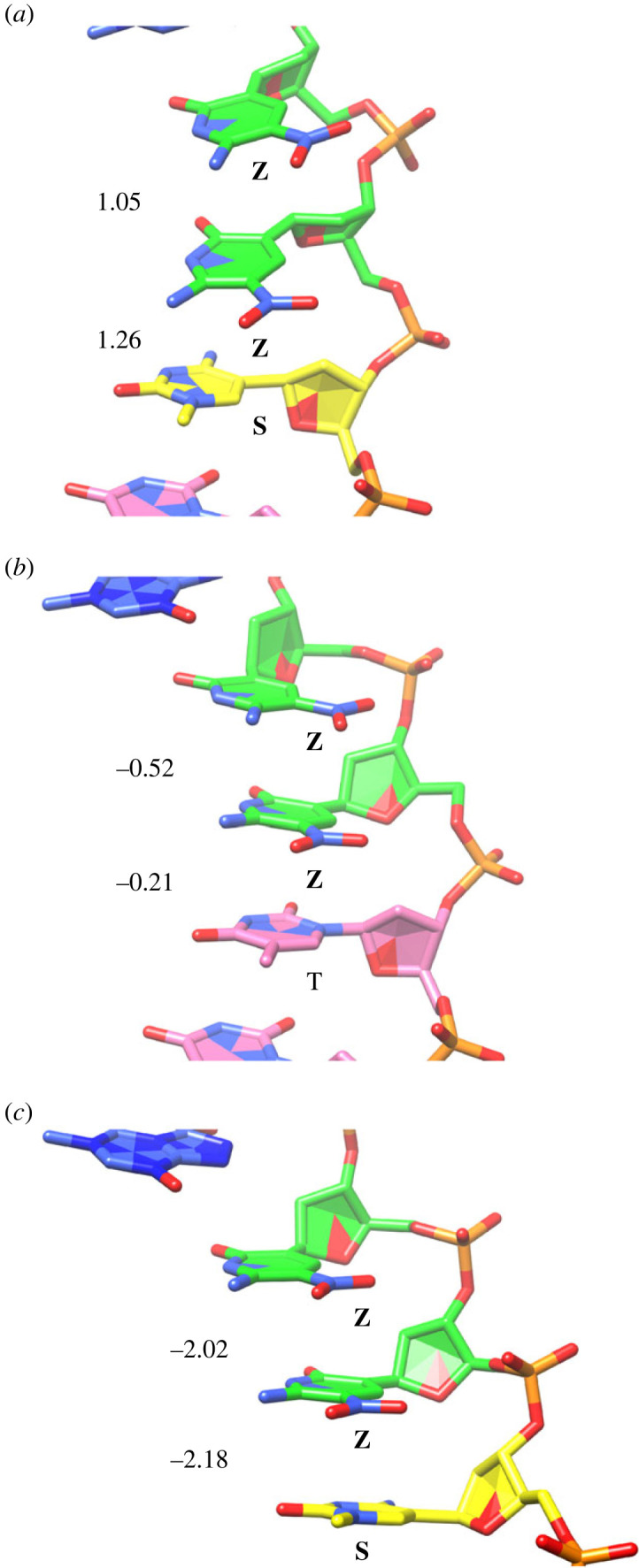
The nitro group in **Z** lacks stacking interactions in the **SZZPBS** structure (*a*) but has some intermediate stacking interactions in **ZZPBS** (*b*). We refer to this as intermediate as it is not fully stacked as in A-form DNA (*c*) but is significantly more stacked than in (*a*). Nucleotides are colour-coded, with **Z** green, **P** blue, **S** yellow and T pink. In (*a*), the sequence 5′-TSZZ corresponds to positions 2–5 in **SZZPBS**, in (*b*) 5′-TTZZP corresponds to positions 2–6 in **ZZPBS**, and in (*c*) 5′-SZZP corresponds to positions 3–6 in an A-form structure of **SZZPBS** (reported elsewhere). Slide values for the dinucleotide steps are shown on the left. This finding suggests that stacking of the nitro group in **Z** confers additional structural variation in ALIEN DNA. (Online version in colour.)

As noted above, there are variations in the roll and slide values involving the **Z:P** pair at position 4 (i.e. dinucleotides steps 3/4 and 4/5). The first three base pairs in **ZZPBS** and **SZZPBS** superimpose well (figure 3*b*). However, the stacking interactions of the **Z:P** pair at position 4 with the base pair at position 3 differ significantly. In previously determined B-form structures including **Z:P** pairs, the nitro group of **Z** does not stack with the nucleobases above or below. In the **SZZPBS** structure, the nitro group in **Z** is not involved in stacking interactions (figure 6*a*). In the **ZZPBS** structure, we observe **Z** nitro stacking that is intermediate, partially stacked (figure 6*b*), with a slide value of −0.21 versus 1.26 Å in **ZZPBS** versus **SZZPBS,** respectively, but not fully stacked as in the A-form structures with a slide value of −2.18 Å (figure 6*c*). This difference in stacking of the nitro group in **Z** then propagates through the duplex DNA in the **ZZPBS** and **SZZPBS** structures, creating significant differences (figure 3*b*). We suggest that the ability of **Z** to stack in alternative modes through its nitro group confers additional structural variability to ALIEN DNA.

## 4. Experimental procedures

### (a) Synthesis and purification of oligonucleotides containing four natural nucleotides (dG, dA, dC and T) and nucleotides (dZ, dP, dB and dS)

Standard phosphoramidites (Bz-dA, Ac-dC, dmf-dG, dT and d**B**: dmf-isodG-CE phosphoramidite) and controlled pore glass (CPG) having standard residues were purchased from Glen Research (Sterling, VA), and AEGIS phosphoramidites (d**Z**, d**P** and d**S**) were purchased from Firebird Biomolecular Sciences (Alachua, FL). All oligonucleotides containing d**Z**, d**P**, d**B** and d**S** were synthesized on an ABI 394 DNA Synthesizer following standard phosphoramidite chemistry and as previously reported [4]. The CPGs having oligonucleotides were treated with 2.0 ml of 1 M 1,8-diazabicyclo[5.4.0]undec-7-ene (DBU) in anhydrous acetonitrile at room temperature for 24 h to deprotect the *p*-nitrophenylethyl (NPE) group on the d**Z** nucleobase. Then the CPGs were filtered, dried and treated with concentrated ammonium hydroxide at 55°C for 16 h. After the removal of ammonium hydroxide, the oligonucleotides containing d**Z**, d**P**, d**B** and d**S** were purified by ion-exchange HPLC (solution A: 25 mM NaOH; solution B: 25 mM NaOH and 1.0 M NaCl). The fraction was neutralized by adding 2.0 M triethylammonium acetate (TEAA) buffer and the oligonucleotides were desalted using Sep-Pac® Plus C18 cartridges (Waters). Chromatograms for the three oligonucleotides following HPLC purification are shown in the electronic supplementary material.

### (b) Crystallization of ALIEN DNA

The oligonucleotides 5′-CTT**BPPBBSSZZS**AAG (**BPPBB**), 5′-CTT**SSPBZPSZBB**AAG (**SSPBZ**) and 5′-CTT**ZZPBSBSZPP**AAG (**ZZPBS**) were dissolved in 10 mM 4-(2-hydroxyethyl)-1-piperazineethanesulfonic acid (HEPES) pH 7.0, 10 mM MgCl_2_, annealed at 70°C for 20 min and then slowly cooled to room temperature at a concentration of 2.5 mM duplex. Host–guest crystals of the oligonucleotide were obtained from vapour diffusion hanging drops containing 1 µL of a complex containing 0.43 mM N-terminal fragment of Moloney murine leukemia virus reverse transcriptase and 0.86 mM duplex DNA, and 1 µL of precipitant solution containing 5 mM magnesium acetate, 0.05 M 2,2′,2′′-nitrilotriacetic acid (ADA) pH 6.5 and 9% polyethylene glycol (PEG) 4000. The counter-ion for all buffers used in crystallographic experiments was Na^+^. The drops contained microseeds generated from host–guest crystals of a natural 16-mer duplex sequence diluted in the precipitant solution. Crystals grew overnight at 20°C and reached optimal size in approximately 2–3 days.

### (c) Data collection and structure determinations

Data were collected for all of the host–guest complex crystals at the Advanced Photon Source, LRL-CAT, beamline 31-ID at a wavelength of 0.97933 Å (table 1). The structures were phased by molecular replacement in PHASER [32] using the protein model alone, avoiding any bias for the electron density associated with the DNA. The starting model was refined in REFMAC [33], and waters were added. The DNA model was then built in iterative cycles of building in COOT [25] and refinement in PHENIX [26] starting with the addition of the first three base pairs. The entire DNA model was initially built as a natural model with T used for **S** or **Z** and G for **B** or **P**. Once the structure of the natural 8 bp duplex had been refined, a single-strand 16-mer was created. The phosphodiester connection between nucleotides 8 and 9 was adjusted, and the model was then refined. The correct unnatural sequence was then built. *F*_o_ − *F*_c_ electron density maps had positive peaks for the missing oxygens associated with the nitro groups in **Z**. Parameter files were created for **S, Z, P** and **B** and used to refine the final model.

### (d) Database for comparative analysis of DNA parameters

We performed an advanced search for A-form DNA within the RCSB database and selected structures deposited between the years 1991 and 2021 having a resolution of 2.0 Å or higher (electronic supplementary material, table S2). We compiled the DNA structures in B-form from host–guest complex structures all crystallized in the same lattice (electronic supplementary material, table S3). These structures were all determined at resolutions ranging from 1.5 to 2.35 Å. We analysed the structures using the web.x3DNA.org database [29] and compiled all of the structural parameters. From each structure, we omitted the modified natural base pairs, such as pairs with methylated or uracil bases, CTT-GAA base pairs interacting with protein, and unnatural base pairing such as purine–purine or pyrimidine–pyrimidine pairs. This yielded a list of 31 structures of A- and 29 structures of B-form. We compiled structural parameters for all natural base pairs and base pair steps.

## Data Availability

We report three crystal structures in this paper. The structures have been deposited in the RCSB.pdb. PDB codes for the structures are 7UYP, 7UYO and 7UYN. The data are provided in the electronic supplementary material [34].
